# Advances in biology, diagnosis and treatment of DLBCL

**DOI:** 10.1007/s00277-024-05880-z

**Published:** 2024-07-17

**Authors:** Yuanfei Shi, Yi Xu, Huafei Shen, Jie Jin, Hongyan Tong, Wanzhuo Xie

**Affiliations:** 1 Department of Hematology, The First Affiliated Hospital, Zhejiang University School of Medicine, No. 79 Qingchun Road, Hangzhou, 310003 Zhejiang China; 2 International Health Care Center, The First Affiliated Hospital, Zhejiang University School of Medicine, Hangzhou, Zhejiang China

**Keywords:** Targeted Therapies, Classification, Genetics, Diffuse large B-cell lymphoma

## Abstract

Diffuse large B-cell lymphoma (DLBCL), with approximately 150,000 new cases worldwide each year, represent nearly 30% of all cases of non-Hodgkin lymphoma (NHL) and are phenotypically and genetically heterogeneous. A gene-expression profile (GEP) has identified at least three major subtypes of DLBCL, each of which has distinct clinical, biological, and genetic features: activated B-cell (ABC)-like DLBCL, germinal-center B-cell (GCB)-like DLBCL, and unclassified. Different origins are associated with different responses to chemotherapy and targeted agents. Despite DLBCL being a highly heterogeneous disease, more than 60% of patients with DLBCL can be cured after using rituximab combined with cyclophosphamide, doxorubicin, vincristine, and prednisone (R-CHOP) to inhibit the growth of cancer cells while targeting the CD20 receptor. In recent decades, the improvement of diagnostic levels has led to a refinement classification of DLBCL and the development of new therapeutic approaches. The objective of this review was to summarize the latest studies examining genetic lesions and therapies for DLBCL.

## Introduction

Approximately 60% of patients with diffuse large B-cell lymphoma (DLBCL), the most common lymphoid malignancy in adults, can be cured with anti-CD20 antibody in combination with cyclophosphamide, doxorubicin, vincristine and prednisone (R-CHOP) [1]. The past few decades have seen numerous targeted therapies discovered, but many patients relapse or die due to their complications. In approximately one-third of patients treated with standard R-CHOP regimens, DLBCL remains the most challenging clinical problem [2, 3]. Due to the heterogeneity of this disease, the treatment effect is limited. In recent years, modern genome-wide molecular analysis of DLBCL has revealed multiple altered pathways associated with tumor development and metastasis, including responses to chemotherapy. Understanding the heterogeneity of this disease will be helpful to further improve treatment outcomes. With these methods, diagnostics and prognostic markers will be developed that are more accurate and reliable, providing opportunities for the development of precision medicine strategies aimed at addressing oncogenic addictions specific to each subtype of lymphoma. Here we summarize the latest data and discuss the genetics and therapies of DLBCL and the new agents in the frontline treatment of DLBCL.

### Subtypes of DLBCL

The World Health Organization (WHO) has updated its classification of haematopoietic and lymphoid tissues for the 5th edition, a B-cell lymphoid proliferations and lymphomas (Table 1) [4]. Most DLBCLs arise de novo, but they can also originate from indolent lymphomas, such as follicular lymphoma (FL) [5–8], chronic lymphocytic leukemia (CLL), or small lymphocytic lymphoma (SLL) [9, 10]. As a secondary disease, DLBCL can also occur in patients who have received solid organ transplants or who are suffering from human immunodeficiency virus (HIV) [11–13].

**Table 1:** WHO classification of haematolymphoid tumours, 4th and 5th edition: B-cell lymphoid proliferations and lymphomas

WHO Classification, 5th edition	WHO Classification, revised 4th edition
Tumour-like lesions with B-cell predominance	
Reactive B-cell-rich lymphoid proliferations that can mimic lymphoma	Not previously included
IgG4-related disease	Not previously included
Unicentric Castleman disease	Not previously included
Idiopathic multicentric Castleman disease	Not previously included
KSHV/HHV8-associated multicentric Castleman disease	Multicentric Castleman disease
Precursor B-cell neoplasms	
B-cell lymphoblastic leukaemias/lymphomas	
B-lymphoblastic leukaemia/lymphoma, NOS	Same
B-lymphoblastic leukaemia/lymphoma with high hyperdiploidy	B-lymphoblastic leukaemia/lymphoma with hyperdiploidy
B-lymphoblastic leukaemia/lymphoma with hypodiploidy	Same
B-lymphoblastic leukaemia/lymphoma with iAMP21	Same
B-lymphoblastic leukaemia/lymphoma with BCR::ABL1 fusion	B-lymphoblastic leukaemia/lymphoma with t(9;22)(q34;q11.2); BCR-ABL1
B-lymphoblastic leukaemia/lymphoma with BCR::ABL1-likefeatures	B-lymphoblastic leukaemia/lymphoma, BCR-ABL1-like
B-lymphoblastic leukaemia/lymphoma with KMT2Arearrangement	B-lymphoblastic leukaemia/lymphoma with t(v;11q23.3); KMT2A-rearranged
B-lymphoblastic leukaemia/lymphoma with ETV6::RUNX1 fusion	B-lymphoblastic leukaemia/lymphoma with t(12;21)(p13.2;q22.1); ETV6-RUNX1
B-lymphoblastic leukaemia/lymphoma with ETV6::RUNX1-likefeatures	Not previously included
B-lymphoblastic leukaemia/lymphoma with TCF3::PBX1 fusion	B-lymphoblastic leukaemia/lymphoma with t(1;19)(q23;p13.3); TCF3-PBX1
B-lymphoblastic leukaemia/lymphoma with IGH::IL3 fusion	B-lymphoblastic leukaemia/lymphoma with t(5;14)(q31.1;q32.1); IGH/IL3
B-lymphoblastic leukaemia/lymphoma with TCF3::HLF fusion	Not previously included
B-lymphoblastic leukaemia/lymphoma with other defined genetic abnormalities	Same
Mature B-cell neoplasms	
Pre-neoplastic and neoplastic small lymphocytic	
proliferations	
Monoclonal B-cell lymphocytosis	Same
Chronic lymphocytic leukaemia/small lymphocytic lymphoma	Same
(Entity deleted)	B-cell prolymphocytic leukaemia
Splenic B-cell lymphomas and leukaemias	
Hairy cell leukaemia	Same
Splenic marginal zone lymphoma	Same
Splenic diffuse red pulp small B-cell lymphoma	Same
Splenic B-cell lymphoma/leukaemia with prominent nucleoli	Not previously included (encompassing hairy cell leukaemia variant and some cases of B-cell prolymphocytic leukaemia)
Lymphoplasmacytic lymphoma	
Lymphoplasmacytic lymphoma	Same
Marginal zone lymphoma	
Extranodal marginal zone lymphoma of mucosa-associated lymphoid tissue	Same
Primary cutaneous marginal zone lymphoma	Not previously included (originally included under “extranodal marginal zone lymphoma of mucosa-associated lymphoid tissue”)
Nodal marginal zone lymphoma	Same
Paediatric marginal zone lymphoma	Same
Follicular lymphoma	
In situ follicular B-cell neoplasm	In situ follicular neoplasia
Follicular lymphoma	Same
Paediatric-type follicular lymphoma	Same
Duodenal-type follicular lymphoma	Same
Cutaneous follicle centre lymphoma	
Primary cutaneous follicle centre lymphoma	Same
Mantle cell lymphoma	
In situ mantle cell neoplasm	In situ mantle cell neoplasia
Mantle cell lymphoma	Same
Leukaemic non-nodal mantle cell lymphoma	Same
Transformations of indolent B-cell lymphomas	
Transformations of indolent B-cell lymphomas	Not previously included
Large B-cell lymphomas	
Diffuse large B-cell lymphoma, NOS	Same
T-cell/histiocyte-rich large B-cell lymphoma	Same
Diffuse large B-cell lymphoma/ high grade B-cell lymphoma with MYC and BCL2 rearrangements High-grade B-cell lymphoma with MYC and BCL2 and/or BCL6 rearrangements	
ALK-positive large B-cell lymphoma	Same
Large B-cell lymphoma with IRF4 rearrangement	Same
High-grade B-cell lymphoma with 11q aberrations	Burkitt-like lymphoma with 11q aberration
Lymphomatoid granulomatosis	Same
EBV-positive diffuse large B-cell lymphoma	EBV-positive diffuse large B-cell lymphoma, NOS
Diffuse large B-cell lymphoma associated with chronic inflammation	Same
Fibrin-associated large B-cell lymphoma	Not previously included (Previously considered a subtype of DLBCL associated with chronic inflammation)
Fluid overload-associated large B-cell lymphoma	Not previously included
Plasmablastic lymphoma	Same
Primary large B-cell lymphoma of immune-privileged sites	Not previously included, encompassing primary DLBCL of the CNS in revised 4th edition (plus primary large B-cell lymphoma of the vitreoretina and primary large B-cell lymphoma of the testis)
Primary cutaneous diffuse large B-cell lymphoma, leg type	Same
Intravascular large B-cell lymphoma	Same
Primary mediastinal large B-cell lymphoma	Same
Mediastinal grey zone lymphoma	B-cell lymphoma, unclassifiable, with features intermediate between DLBCL and classic Hodgkin lymphoma
High-grade B-cell lymphoma, NOS	Same
Burkitt lymphoma	
Burkitt lymphoma	Same
KSHV/HHV8-associated B-cell lymphoid proliferations and lymphomas	
Primary effusion lymphoma	Same
KSHV/HHV8-positive diffuse large B-cell lymphoma	HHV8-positive diffuse large B-cell lymphoma, NOS
KSHV/HHV8-positive germinotropic lymphoproliferative disorder	HHV8-positive germinotropic lymphoproliferative disorder
Lymphoid proliferations and lymphomas associated with immune deficiency and dysregulation	
Hyperplasias arising in immune deficiency/dysregulation	Not previously included, encompassing non-destructive post-transplant lymphoproliferative disorders, among others
Polymorphic lymphoproliferative disorders arising in immune deficiency/dysregulation	Not previously included, encompassing polymorphic posttransplant lymphoproliferative disorders, other iatrogenic immunodeficiency-associated lymphoproliferative disorders, among others
EBV-positive mucocutaneous ulcer	Same
Lymphomas arising in immune deficiency / dysregulation	Not previously included, encompassing monomorphic posttransplant lymphoproliferative disorders, classic Hodgkin lymphoma posttransplant lymphoproliferative disorders, lymphomas associated with HIV infection, among others
Inborn error of immunity-associated lymphoid proliferations and lymphomas Lymphoproliferative diseases associated with primary immune disorders	
Hodgkin lymphoma	
Classic Hodgkin lymphoma	Same
Nodular lymphocyte predominant Hodgkin lymphoma	Same
Plasma cell neoplasms and other diseases with paraproteins	
Monoclonal gammopathies	
Cold agglutinin disease	Not previously included
IgM monoclonal gammopathy of undetermined significance	Same
Non-IgM monoclonal gammopathy of undetermined significance	Same
Monoclonal gammopathy of renal significance	Not previously included
Diseases with monoclonal immunoglobulin deposition	
Immunoglobulin-related (AL) amyloidosis	Primary amyloidosis
Monoclonal immunoglobulin deposition disease	Light chain and heavy chain deposition disease
Heavy chain diseases	
Mu heavy chain disease	Same
Gamma heavy chain disease	Same
Alpha heavy chain disease	Same
Plasma cell neoplasms	
Plasmacytoma	Same
Plasma cell myeloma	Same
Plasma cell neoplasms with associated paraneoplastic syndrome	Same (Except AESOP syndrome not previously included)
-POEMS syndrome	
-TEMPI syndrome	
-AESOP syndrome	

### Gene expression profiling

DLBCL can be divided into two main subgroups based on its cell-of-origin (COO): germinal center B-cells (GCBs) and non-GCBs. Different subgroups represent different molecular characteristics and clinical behavior [14]. Based on transcriptome sequencing, researchers found that there were different gene mutations among different subtypes of DLBCL [15–17]. The analyses have been based on COO analysis, immunohistochemistry (IHC) algorithms, and gene expression profiling (GEP) techniques, all indicating that DLBCL patients have a more common non-GCB phenotype, accounting for 59% to 75% of cases compared to 50% in patients with advanced-stage disease [18–22]. ABC subgroup patients with MYD88, CD79B, and NOTCH1 mutations have a poorer prognosis than patients with other mutations [16]. DLBCL subgroups with EZH2 mutations and BCL-2 translocations are associated with worse outcomes in GCB-DLBCL patients [16]. Similarly, double-hit/triple-hit (DH/TH) (~ 7%) is a type of high-grade B-cell lymphoma (HGBL), that has MYC, BCL-2 (~ 20%) or/and BCL-6(~ 14%) rearrangements [18, 22–24]. Due to the clear genetic and biological differences between ABC and GCB DLBCL, patients with ABC have a worse prognosis than those with GCB when treated with R-CHOP as a first-line treatment [25–31]. The treatments and outcomes for DLBCL subtypes see Table 2.

**Table 2:** Treatments and outcomes for DLBCL subtypes

COO subtype	Treatment	3y-OSR	5y-OSR	3y-EFS	5y-PFS	Ref
GCB-DLBCL	R-CHOP	87%	80%		65%	[32]
	G-CHOP			94%	71%	[33]
ABC-DLBCL	R-CHOP	60%	45%		56%	[32]
	G-CHOP			58%	54%	[33]

*OSR* 5- or 3-year overall survival rate, *EFS* event-free survival, *PFS* Progression free survival

**Fig. 1:**
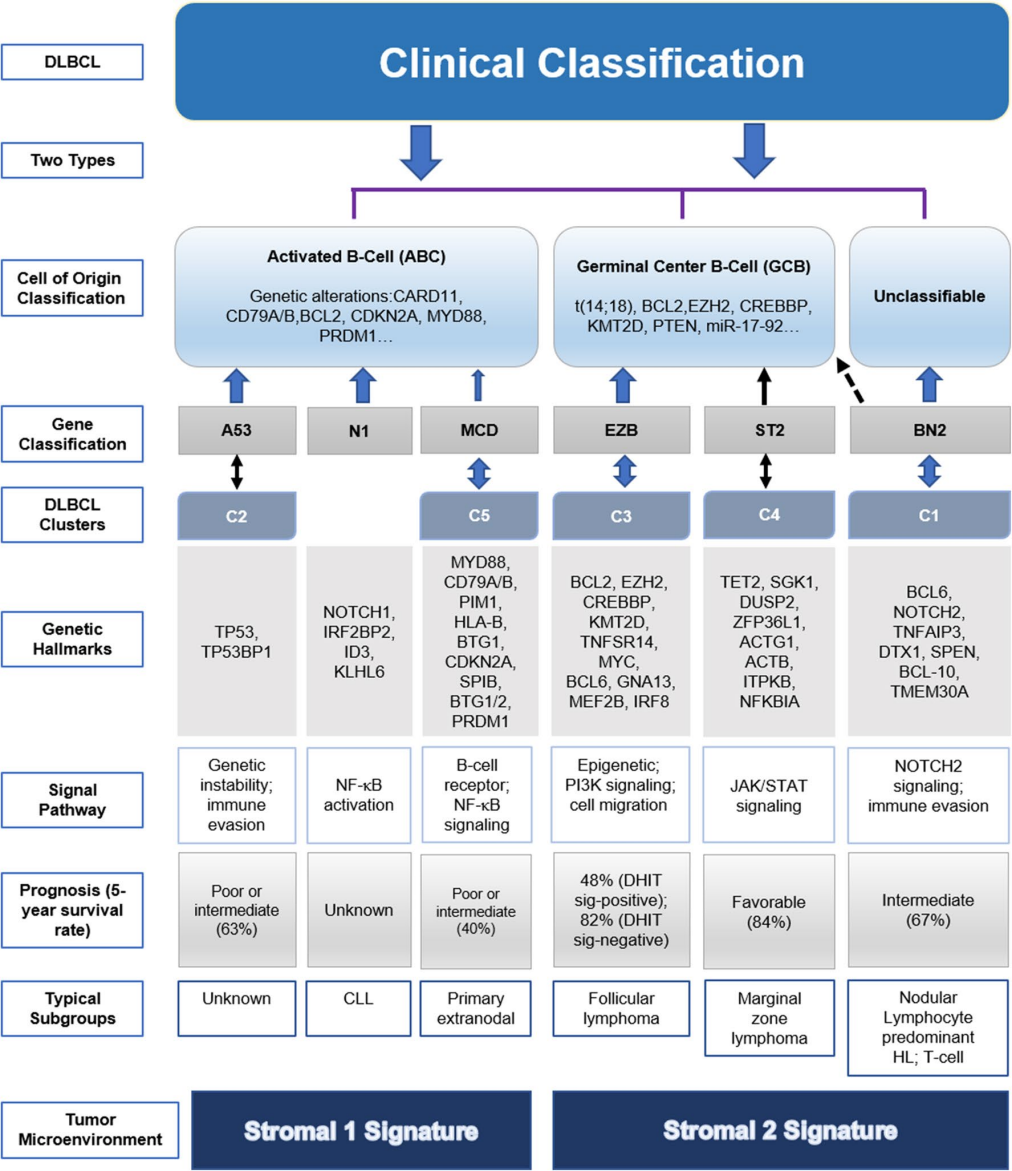
Outcomes and classification of diffuse large B-cell lymphoma (DLBCL), risk factors, and biologic features

The use of microarrays to analyze gene expression profiles is another method to classify DLBCL in relation to different aspects of the disease’s biology. Tumor microenvironments (TMEs) are characterized by the differential expression of genes involved in oxidative phosphorylation and B-cell receptor (BCR) signaling as well as Molecular heterogeneity in diffuse large B-cell lymphoma and its implications in clinical diagnosis and treatment the inflammatory response of the host [34]. The pathogenesis of DLBCL involves somatic mutations that include chromosomal aberrations, translocations, and copy number changes in specific chromosomal regions. By RNA sequencing, a somatic mutation frequency of 3–6 mutations is observed, which is more common than renal cell carcinoma and acute leukemia (AL) but much less than solid tumors, such as melanomas or lung cancers (> 10 mutations) [35–37]. Each lymphoma has 20 to 400 different gene mutations that affect the coding DNA sequences [37–39]. Different gene mutations exist in different subtypes of DLBCL, which are usually related to the prognosis of patients, and some mutations only occur in specific subtypes (Fig. 1).

As gene next-generation sequencing is conducted more frequently in clinical examinations, the subgroups defined at the genetic level largely direct prognosis and therapeutic regimens. Subgroups based on genetics, although partially coincident with COO subgroups, show more accuracy. The BN2 subgroups contain 41% ABC and 19% GCB types as reported by Roland Schmitz and feature damage to NOTCH pathways; thus, BTK inhibitors can be used, especially ibrutinib [16]. Additionally, in the MCD, BN2 and EZB subgroups, especially PI3K signaling inhibitors can make results. Furthermore, the genetic subtypes indicate prognosis. Schmitz R and his workmates used the genetic algorithm, which did not use clinical information found that the four subtypes differed significantly in progression-free survival, the 5-year survival rates in the MCD, N1, BN2 and EZB subgroups are 26%, 36%, 65% and 68%, respectively [16]. G. W. Wright proposed extra ST2 and A53 subgroups, and discriminated EZB subgroups by whether MYC expressed as the significantly different survival results occurring [17]. A study that recruited 105 patients whose pathological gene sequencing data were available showed poor prognosis in the N1 and A53 subgroups. This more accurate classification can bring more value to both therapy and prognosis.

### ABC subtype lesions

#### B-cell differentiation

One of the main mechanisms underlying the pathogenesis of DLBCL is the normal process of GCB during the development or occurrence of gene mutations. Deregulating BCL-6, the main regulator of GCB differentiation, directly affects this process. Various cellular functions are regulated by BCL-6, including DNA damage responses, cell cycle progression, and signal transduction [29, 40–46]. Chromosomal translocations affect BCL-6 (3q27) more frequently in ABC DLBCL than in GCB DLBCL, resulting in the deregulation of BCL-6 expression. BCL-6 expression is considered to be related to improved outcomes, reflecting the prognosis of GCB [47–50]. Some researchers have found that BCL-6 translocations affect the prognosis of patients, but relevant studies have not been fully confirmed [51–53].

The deletions of DLBCL often occur in 6q23 and 6q21 [54–57]. PRDM1 (PR domain containing 1, with ZNF domain) is a transcriptional repressor important for the terminal differentiation of B-cells into plasma cells. Alterations in BLIMP1 only occur in ABC subgroups. In addition, BCL-6 is one of the most important repressors of BLIMP1, and changes in BCL-6 can also affect BLIMP1. These findings indicate that translocations or mutations contribute to the development of ABC subgroups [57]. Chromosomal translocations and genomic gains in ABC subgroups target SPIB (19q13.3-q13.4) [58, 59]. BLIMP also targets SPIB, with high expression in ABC subgroups compared to GCB subgroups [60].

### BCR and NF-κB signaling

Most changes in the ABC subgroup are caused by activation of the NF-κB signaling pathway. Genetic lesions among the different genes activate the NF-κB pathway, illustrating the many pathways that cause NF-κB activation in normal GCBs. Somatic mutations and deletions inactivate a relatively small number of genes, including TNFAIP3 (∼30%), MYD88 (∼30%), CARD11 (∼10%), TRAF5 (∼5%), CD79B, CD79A (∼20%) and TRAF2 (∼3%), while RANK (∼8%) is activated largely because of somatic mutations [37, 38, 61–64]. Overall, 20%-30% of DLBCLs have TNFAIP3 mutations, especially in ABC subgroups. Lymphoma cells inactivate TNFAIP3 and also negatively regulate NF-κB [49, 65, 66]. MYD88 mutations are present in approximately 30% of cases of ABC subgroups [64]. Among the MYD88 mutations, the L256P mutation is the most common mutation that simultaneously activates the JAK-STAT3 pathway [67, 68]. A mutation of CARD11 in GCB-subgroups can activate NF-κB even in the absence of antigen receptor signals (such as CD40-CD40L). Mutations in CD79A and CD79B are the most common ABC subtypes, and they are important components of BCR. CD79B and CD79A induce surface BCR expression through their effects on ITAM tyrosine residues [63]. BCL-6 and FOXP1 are the most common dysregulated genes of ABC subgroups. Additionally, NFKBIZ contributes to lymphomagenesis and is involved in the NF-κB and STAT3 pathways [69].

### GCB subtype lesions

#### BCL-2 chromosomal translocation

In DLBCL, it is very common deregulate BCL-2 (18q21). The t (14;18) (q32; q21) translocation connects BCL-2 to the immunoglobulin heavy chain (IGHV) gene enhancer (14q32), resulting in BCL-2 deregulation [70–72]. The t (14;18) translocation occurs in 30%-40% of GCB subgroups [70], but it is not unique to DLBCL; rather, it occurs in 90% of follicular lymphomas. In the GCB subgroups, follicular lymphoma rarely presents in those younger than 18 years of age [73]. In the ABC subgroups, BCL-2 is rarely translocated (30%-40% of the cases), but it is more prone to gain or be amplified than in the GCB subgroups (15%) [74]. In GCB DLBCL, the promoter of BCL-2 is also frequently mutated [40, 61, 75], which is related to the presence of t (14;18). Although the prognosis of patients may be related to BCL-2 mutations and the treatment regimen adopted, technical biases might also impede the treatment effect [74, 76, 77]. Recently, two different large studies compared the effect of t (14;18) and BCL-2 on patient outcomes with R-CHOP, but only one study indicated that t (14;18) is related to poor outcomes in GCB patients [72]. Based on the results of both studies, BCL-2 is associated with poor prognosis in GCB DLBCL, but not in ABC DLBCL [72]. Contrary to previous studies, BCL-2 is only a poor prognostic factor for ABC subgroups [78].

### EZH2

By sequencing and DNA profiling, EZH2 was found to be one of the most commonly mutated gene, occurring in approximately 6%-14% of DLBCL [39, 79–81]. It appears almost exclusively in the GCB subtypes, especially with BCL-2 translocations [81]. In 20% of GCB subgroups, EZH2 mutations are associated with t (14;18), but they are rarely seen in ABC DLBCL. Investigators believe that EZH2 inhibitors are considered promising preclinical data [82–85], and early relevant clinical trials are ongoing. DLBCL is accompanied by other gene mutations when chromatin modification occurs. Because of the low mutation rate and differences in the studied series, it is difficult to estimate the association with any specific subtype. There are several genes linked to DLBCL, including MLL2 (KMT2D) (22%-32% DLBCL), CREBBP (18%-20%), EP300 (5%-10%), and MLL3 (KMT2C) (15%) [79].

### The lymphoma microenvironment (LME) can be divided into four distinct categories

Microenvironment cells and extracellular matrix (ECM) are responsible for external stimuli in the lymphoma niche, according to data obtained from lymphoma patients and animal models, leading to the development and progression of the disease, as well as the response to treatment [25, 86–89]. Due to bidirectional interactions between lymphoma cells and their microenvironment [90, 91], the complexity of the DLBCL microenvironment has yet to be defined. Although the DLBCL microenvironment has attracted increasing attention [25], people often only give attention to the disease itself during treatment and ignore the important role of the microenvironment [14]. DLBCL microenvironments vary in composition and functionality based on the gene expression profiles of thousands of patients. Twenty-five functional gene expression signatures (F^GES^) were discovered, reflecting either distinct cellular subtypes or noncellular components of the tumor microenvironment and activation of canonical signaling pathways in biological processes [92]. Nikita Kotlov et al. reported that the LME in DLBCL integrates characteristics of the microenvironment and malignant cells into the prognosis. We named the four distinct LMEs as follows: “germinal center-like” due to the presence of F^GES^ from cell types commonly found in germinal centers (GC); “mesenchymal” (MS) refers to the abundance of F^GES^ within stromal cells and intercellular matrix; “inflammatory” (IN) indicates F^GES^ that are found in inflammatory cells or pathways; and “depleted” (DP) LMEs are characterized by an overall lower presence of F^GES^ derived from the microenvironment [92]. Transcriptomic studies have found that the microenvironment correlates with disease biology [25, 30, 34]. Initial research focused on identifying differences in gene expression profiles among tumor samples [30, 34]. They extracted microenvironment signatures from transcriptomics to identify microenvironment cells in the transcriptome [93], and four distinct microenvironments reflecting unique biological characteristics and clinical behavior were proposed. As a result of these newly developed categories, we have identified distinct clinical behaviors among genetically similar DLBCLs and promising therapeutic targets [92].

### Immunohistochemistry

The emergence of immunohistochemistry has met the increasing demand for personalized medicine, and the utility of applying complex genomics to clinical practice is clear. In recent years, according to the morphological review of the WHO classification of hematopoietic and lymphoid tissue tumors in 2017 and 2022, IHC is an important method for diagnosing and stratifying DLBCL [94]. Although there are many classification standards for DLBCL, the WHO mostly adopts the Hans criteria classification [95] (Table 3). IHC has always been considered one of the criteria for diagnosing DLBCL. Nevertheless, the Hans diagnostic criteria are approximately 80% consistent with gene expression profiling derived ABC-DLBCL and GCB-DLBCL classifications [95]. However, the accuracy of IHC diagnosis is challenged by GEP because there is an operation change of dyeing intensity in IHC. Nevertheless, with the widespread application of GEP and multi-genome platform analysis, IHC as an auxiliary tool for verifying genes is becoming increasingly important [96]. IHC can evaluate the degree of tumor infiltration [97], tumor microenvironment proteins [98], expression of tumor-promoting and tumor suppressor genes [99], and others. However, as the interpretation of IHC results varies by person, it is difficult to use it as the only criterion for disease diagnosis. With the advent of genomics and other new computational tools, the importance of IHC has been gradually weakened [96]. Another important role of IHC is to evaluate the prognosis of patients, especially in patients with double-expression, that is, ≥ 40% MYC and ≥ 50% BCL-2 are simultaneously expressed in lymphoma cells [100]. The researchers established a correlation with the double expression lymphoma score, which can effectively predict the inferior outcome of these patients; other studies have also supported this idea [101–103].

**Table 3:** Several classification methods of DLBCL subtypes[1]

Classification	Antibodies	Comments
Hans et al. [95]	CD10, BCL-6, MUM1	≥ 30% staining to be considered positive
Choi et al. [104]	CD10, BCL-6, MUM1, GCET, FOXP1	≥ 80% GCET, FOXP1, MUM1; ≥ 30% staining for CD10 and BCL6
Muris et al. [105]	BCL-2, CD10, MUM1	BCL2 ≥ 50% and CD10 or BCL6 ≥ 30%
Nyman et al. [106]	MUM1, FOXP1	≥ 30% staining to be considered positive
Natkunam et al. [107]	CD10, MUM1	LMO2 > 30%
Meyer et al. [108]	CD10, GCET1, LMO2	MUM > 30%; FOXP 1 > 80%
Visco et al. [31]	CD10, BCL-6	FOX1 > 10%

### Diagnosis and staging

#### Molecular diagnosis

The molecular classification of DLBCL requires an excisional biopsy and expert hematopathologist review to ensure adequate tissue available for diagnostic assessment [109]. When the excisional biopsy cannot recognize the tumor type, a core biopsy is required [110, 111]. The diagnosis of DLBCL is based on the WHO 2022 criteria [4]. Somatic mutation and intraclonal variation in the V region of the Ig gene are characteristic changes in GCB cells [112, 113]. BCL-6 and CD10 are markers of germinal center B-cells, while IRF4/MUM1 is mainly expressed in the late stage of plasma cell and B-cell development and is a marker of non-GCB [114, 115]. IRF4 is transiently expressed when activated by normal lymphocytes and participates in the proliferative response of B-cells after antigen activation [116–118]. During ABC-type cell proliferation and tumor formation, IRF4 plays an important role in constitutive expression [119, 120]. Therefore, DLBCL-not other specified (DLBCL-NOS) can be classified as GCB according to CD10, BCL-6 and IRF4/MUM1 and non-GCB [121]. GCB can be diagnosed in the following cases: CD10 is positive; CD10 is negative, but BCL-6 is positive and IRF4/MUM1 is negative, and the others are non-GCB [122, 123].

Gene expression analysis showed that the t (14; 18) (q32; q21) translocation involves the BCL-2 gene and is found only in GCB subtypes [124]. The 3q27 translocation involving BCL-6 can be found in 30% ~ 40% of DLBCL cases. The expression of BCL-6 plays a significant role in the development of the germinal center and the response to antigens; thus, it is known as a germinal center marker [125]. It has been reported that BCL-6 can inhibit the expression of PRDM1, which is an important regulatory gene for plasma cell differentiation [126]. In addition, inhibiting the normal downregulation of BCL-6 leads to cell differentiation arrest and continued proliferation, thus leading to tumorigenesis [127]. It has also been shown that abnormal chromosome translocation results in the deregulation of BCL-6, which inhibits the downregulation of BCL-6 expression, causing abnormal expression of BCL-6 in some non-GCB DLBCL subgroups [128].

Aberrant activation of the NF-κB pathway is a feature of ABC subtypes. The activation of NF-κB caused by the excessive activity of IKK leads to rapid IκB degradation by pantothenate proteasome, resulting in NF-κB release and translocation to the nucleus to activate a series of transcription factors. This promotes cell proliferation and inhibits apoptosis, which results in long-term tumor cell survival [129, 130]. Because constitutive activation of IKK is a unique feature of ABC subtypes, NF-κB may be a new potential treatment target for ABC subtypes, and it has been confirmed that inhibition of IKK activity can promote apoptosis of ABC subtypes but not GCB subtypes [131]. In addition, ABC and GCB also have obvious differences in response to IL-4 [132]. IL-4 promotes GCB subtypes to induce high expression of downstream target genes, such as BCL-6, through activating signal transcription activator 6 (STAT6 phosphorylation) and ultimately promotes cell proliferation [133]. This may explain why ABC-DLBCL is not sensitive to cell cycle drugs. We summarize the differences.

between GCB and ABC in Table 4.

**Table 4:** Comparison of differential genes between GCB and ABC subtypes of DLBCL [1]

DLBCL Classification	Gene markers	Recurrent translocations	Most common genomic aberrations	Most common somatic mutations
Germinal Center B-cell (GCB)-like DLBCL	LMO2, MYBL1, BCL-6	t(14;18)(q32;q21) IGHV-BCL2, 20%–	+ 1q, + 2p16 (REL), + 7q, + 12q (MDM2),	Chromatin remodeling (EZH2, MLL2, MEF2B,
	NEK6, TNFRSF9	45%; 8q24 rearrangements involving	+ 13q31 (MIRHG1), − 1p36	EP300, CREBBP), TP53, BCL6 regulatory
		MYC, 20%; 3q27 rearrangement	(TNFRSF14), − 10q23 (PTEN), − 13q34	region and other aberrant somatic
		involving BCL6, 10%	(ING1), − 17p (TP53)	hypermutation targets
Activated B-cell (ABC)-like DLBCL	IRF4, FOXP1, IGHM	3q27 rearrangements involving BCL6,	Trisomy 3 (FOXP1, NFKBIZ), + 18q21	Chromatin remodeling (MLL2, EP300,
	TNFRSF13B, CCND2	25%; 8q24 rearrangements involving	(BCL2, NFATC1), + 19q13 (SPIB),	CREBBP), BCR signaling and NF-κB
		MYC, 5%	− 6q21 (PRDM1), − 6q23 (TNFAIP3),	pathway (TNFAIP3, CARD11, CD79B,
			− 9p21 (CDKN2A), − 17p (TP53	MYD88, TRAF2, TRAF3, TRAF5, MAP3K7,
				TNFRSF11A, ITPKB), PRDM1, BCL6
				regulatory region and other aberrant somatic
				hypermutation targets, TP53

### Other adjunctive diagnoses

In some selected circumstances, bone marrow biopsy (BMB) remains an important diagnostic method for DLBCL. The clinical manifestation, organ function evaluation and Ann Arbor score of patients are also essential as important auxiliary diagnostic methods. PET-CT combines the benefits of PET and contrast-enhanced CT and should therefore be recommended for all DLBCL patients for diagnosis and efficacy evaluation; importantly, it can identify more DLBCL cells than a standard contrast-enhanced CT alone, with PET staging in 5% to 15% of DLBCL [134, 135]. A superior option in Lugano staging recommendations is BMB, which has shown valuable in the PET era [134]. DLBCL is widely diagnosed using PET-CT, which provides high sensitivity and specificity. However, indolent or low-volume disease may go undetected [136]. Thus, BMB is still the most accurate, reliable and irreplaceable diagnostic method for DLBCL.

### Treatment

The R-CHOP regimen can cure 60% of DLBCL patients [137, 138]. However, with the continuous development of diagnosis and treatment technology, more individualized treatment should also be widely used. For elderly patients with poor basic conditions, the chemotherapy cycles and times can be shortened according to the disease location and scope to reduce the chemotherapy risk. The four treatment regimens based on rituximab are the main regimens for the treatment of DLBCL at this stage.

### Combination therapy: chemotherapy plus involved-site radiotherapy (ISRT)

In bulky (≥ 7.5 cm) DLBCL patients, radiotherapy as a treatment for the consolidation phase after chemotherapy can bring benefits to patients. Among non- bulky (< 7.5 cm) DLBCL patients, patients with limited disease duration and smIPI score ≥ 1 received a 3-cycle RCHOP regimen combined with 40–46 Gy doses of radiotherapy. The PFS for 2 and 4 years is 93% and 88%, respectively. 95% OS at 2 years, 92% at 4 years [139]. In another experiment comparing RCHOP and RCHOP-RT, it was found that there was no statistically significant difference in 5-year EFS between the two groups. The R-CHOP group had 89% ± 2.9%, while the R-CHOP combined with RT group had 92% ± 2.4%. The OS of patients receiving R-CHOP treatment alone was 92% (95% CI, 89.5% -94.5%), while R-CHOP-RT was 96% (95% CI, 94.3%-97.7%) (P not significant). Therefore, in non-bulky DLBCL patients, the benefits of chemotherapy combined with radiotherapy are not significant [138]. In addition, some special extranodal DLBCL, such as CNS DLBCL, with primary ocular involvement, localized skin involvement, testicular involvement, etc., are also recommended for radiation therapy during the consolidation phase [140].

### Standard R-CHOP

The R-CHOP regimen was found to be effective in treating DLBCL patients aged 18 to 60 years, with a favorable overall survival (OS) rate after combining rituximab. Seventy-two percent of patients were in stages I to II, and only 3% had a baseline mass greater than 10 cm. Compared with chemotherapy alone, the combination of rituximab improved the OS of patients from 80 to 90% at six years. Patients with a mass size < 5 cm and without other IPI risk factors had the best outcomes, with 95% OS at 6 years [141]. The effectiveness of R-CHOP is equivalent to that of combined modality treatment (CMT), avoiding RT by enhancing systemic therapy compared to whole-course R-CHOP [142, 143]. However, some researchers found that, after 6 to 8 cycles of R-CHOP chemotherapy plus ISRT, the progression-free survival (PFS) and OS of patients were improved [144]. In general, the above results showed that conventional ISRT after whole-course R-CHOP treatment has certain benefits, but the side effects of ISRT are often found decades after treatment. The use of R-CHOP alone for 6 cycles has proven safe and effective in the treatment of DLBCL, especially in patients with high-risk diseases. This type of patient includes: stage I and II (excluding stage II with extensive mesenteric diseases) with or without large masses (≥ 7.5 cm). Clinical practitioners are also striving to tailor treatment programs based on the patient’s conditions to promote the concept of individualized treatment.

### R-CHOP plus ‘X’

**Fig. 2:**
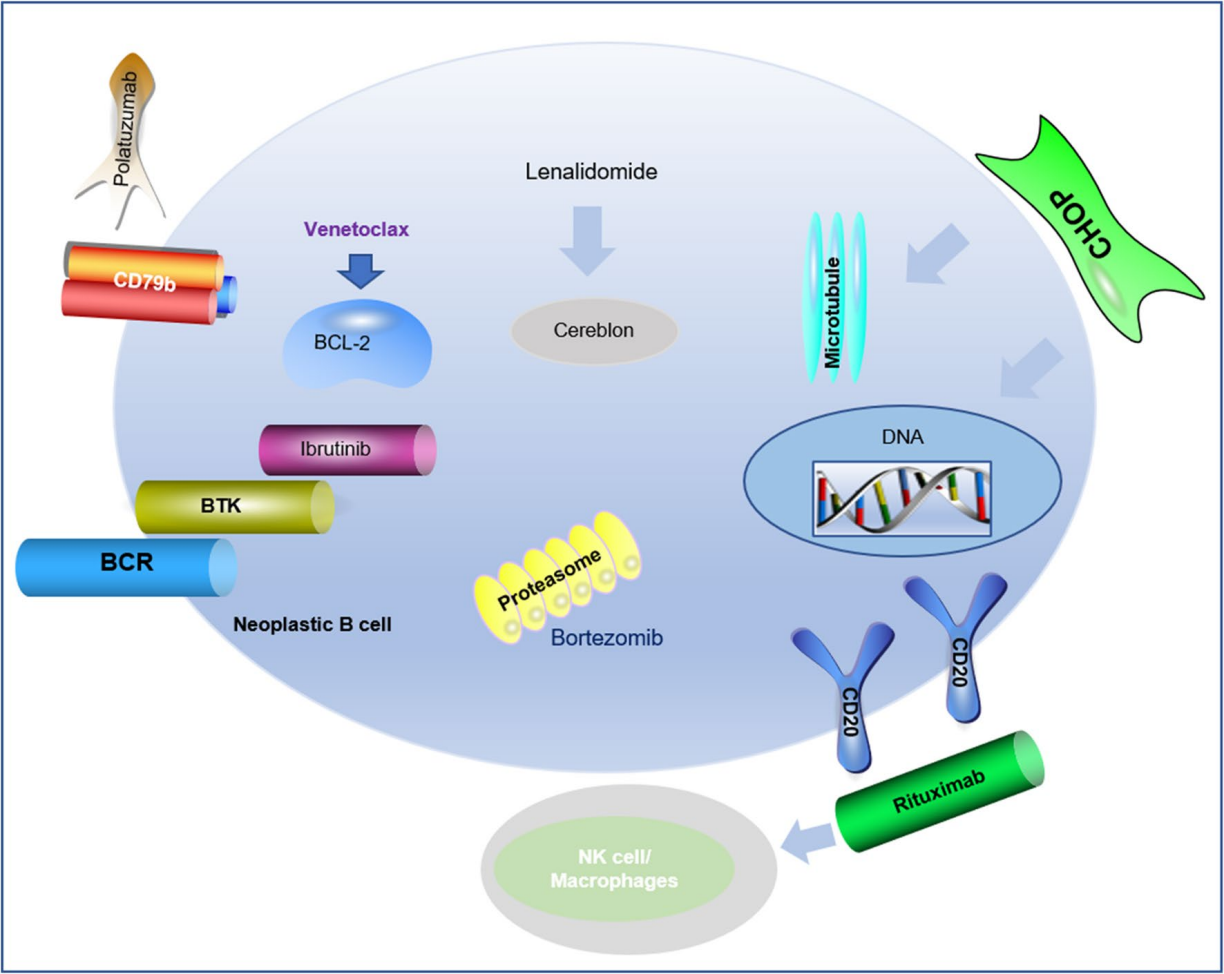
Mechanism of R-CHOP regimen plus ‘X’ drugs in treatment of DLBCL. The backbone R-CHOP has been combined with a number of add-on therapies. The immunomodulatory effect of malidomide is mediated by the regulation of T/NK cells, Venetoclax blocks anti-apoptotic protein BCL-2, Lenalidomide is an immunomodulant agent that blocks cereblon, Bortezomib inhibits proteasomes, Ibrutinib inhibits Bruton Tyrosine Kinase, and Polatuzumab inhibits CD79b

Based on the original standard first-line treatment scheme, R-CHOP + ‘X’ has become increasingly popular with patients over time. Figure 2 illustrates the mechanism by which the R-CHOP regimen plus ‘X’ drugs are used in treating DLBCL.

In phase II single arm trials of lenalidomide with R-CHOP (R2-CHOP), the drug showed promise as a frontline therapy for non-GCB DLBCL [145–147]. Consequently, R2-CHOP was subsequently tested for DLBCL in two randomized studies in comparison to R-CHOP. A phase II randomized clinical trial involving 349 patients demonstrated a positive difference in both OS and PFS for patients with the ABC subtype of DLBCL treated with R2-CHOP [148]. In a phase III trial, consisting of 570 ABC-DLBCL patients, lenalidomide was added with a slightly different schedule from the previous study, although patients with high-risk diseases (IPI score 3 or more) showed a trend favoring R2-CHOP over placebo/RCHOP, the PFS did not improve [149].

The proteasome inhibitor Bortezomib was also unable to improve outcomes over R-CHOP in the phase II PYRAMID study or in the phase III REMoDL-B study. In the latter study, in which patients were also stratified based on their COO, no differences were observed between the two arms [150]. Similarly, when added to R-CHOP (RB-CHOP), the proteasome inhibitor Bortezomib failed to improve outcomes compared to R-CHOP in phase II PYRAMID and phase III REMoDL-B phase III trial, a subsequent study that also stratified patients by COO did not find any differences between the two arms [150]. In patients with double-hit lymphoma, PFS at 30 months is higher after R-CHOP in comparison to RB-CHOP at 58.8%, although this was derived from a post-hoc analysis, and the difference was not statistically significant [151].

Ibrutinib is an oral inhibitor of Bruton’s tyrosine kinase (BTK) and has been approved for several B-cell malignancies, including R/R ABC DLBCL, possibly related to the chronic activation of B-cell receptor and NF-κB patterns which characterize this COO subtype [148]. However, in the phase III PHOENIX trial, ibrutinib plus R-CHOP was compared to placebo + RCHOP, but neither of the primary or secondary endpoints were significantly improved [151]. A pre-planned exploratory analysis identified a significant interaction between treatment and age: when administered to patients under 60 years of age, ibrutinib plus R-CHOP resulted in improved outcomes with manageable safety, but when given to older patients, the addiction to ibrutinib led to adverse effects and compromised treatment administration [151]. With the purpose of ameliorate PHOENIX results, ESCALADE (NCT04529772) is a phase III trial randomized to perform R-CHOP or R-CHOP plus acalabrutinib on young untreated non-GCB DLBCL patients (65 years old), a selective second-generation BTK inhibitor with fewer off-target side effects [151].

#### Pola + R-CHP

Polatuzumab vedotin is an antibody–drug conjugate that combines monoclonal antibodies targeting CD79b, a cell-surface antigen expressed exclusively on mature B cells except plasma cells, with monomethyl auristatin E, a cytotoxic agent. Since 2021, public health insurance systems in Japan have approved and covered polatuzumab vedotin for the treatment of relapsed or refractory DLBCL [151]. Pola + R-CHP (polatuzumab vedotin plus rituximab, cyclophosphamide, doxorubicin, and prednisolone) combination therapy was evaluated in a phase III, multi-institutional, randomized, double-blind, placebo-controlled trial (POLARIX: GO39942). A study showing superiority of Pola + R-CHP over CHOP therapy for previously untreated CD20-positive DLBCL with an IPI score of 2 showed that Pola + R-CHP delivered superior PFS (A risk ratio of 0.73 [95% CI: 0.57–0.95; p = 0.02] was obtained for progression, relapse, or death) as compared with R-CHOP regimens. Despite this, OS did not differ significantly between the groups (hazard ratio for death = 0.94 [95%CI: 0.65–1.37; p = 0.75]). According to data from the POLARIX trial and other studies, Pola + R-CHP was approved by the Japan Ministry of Health and Welfare in August 2022 [151].

#### PET-adapted therapy

PET was used as an important auxiliary tool for the diagnosis of DLBCL, which filled the gap in imaging, with the following three objectives staging, prognosis evaluation, and response to treatment. Disease staging by PET can find additional sites of lesions in 35% of patients, and 12% of patients have higher stages [152]. A retrospective study of prognosis found that 56% of the positive predictive value had an IPI < 3, compared with 80% for patients with an IPI ≥ 3. Using PET to monitor disease recurrence, the accuracy rate can reach more than 95% [153]. Generally, negative PET indicates a good prognosis, and CT re-examination may not be required in a short time [154]. The treatment plan of the British Columbia Cancer Agency (BCCA) for DLBCL patients is that the patients receive three cycles of R-CHOP treatment achieving a complete remission (CR) by PET and then receive an additional cycle of R-CHOP treatment. To better clear residual lesions, ISRT is also acceptable. Approximately 80% of the studied population had at least one risk factor for stage-modified IPI (smIPI). The 3-year PFS and 3-year OS of patients with negative interim PET (iPET) results were 92% and 96%, respectively, while the 3-year PFS and OS of patients with positive iPET results were 60% and 83%, respectively [155, 156]. This study showed that the time and related toxicity of chemoimmunotherapy can be reduced by using iPET to evaluate the therapeutic effect, while patients with iPET positivity still need to optimize treatment. Another study further evaluated whether R-CHOP at 6 cycles after PET imaging was better than that at 4 cycles. The initial treatment of DLBCL patients with R-CHOP was two cycles, those with iPET-negative tumors received only four cycles, and those with iPET-positive tumors received a total of six cycles. After 5 years of follow-up, all patients treated with R-CHOP achieved 92% PFS in the experimental group and 89% PFS in the standard group at 3 years [157]. Therefore, it can effectively evaluate the patient’s condition, select different treatment regimens, shorten the treatment cycle and reduce the treatment risk. Their research also discovered the role of other PET-derived biomarkers, such as metabolic tumor volume, which are predictive of PFS [158] and OS [159]. Research from Wyndham H. Wilson et al. Showed that ibrutinib with R-CHOP could increase event-free survival (EFS) of patients with MCD DLBCL from 48% to 69.6% [160].

#### Treatment of relapsed refractory DLBCL

**Fig. 3:**
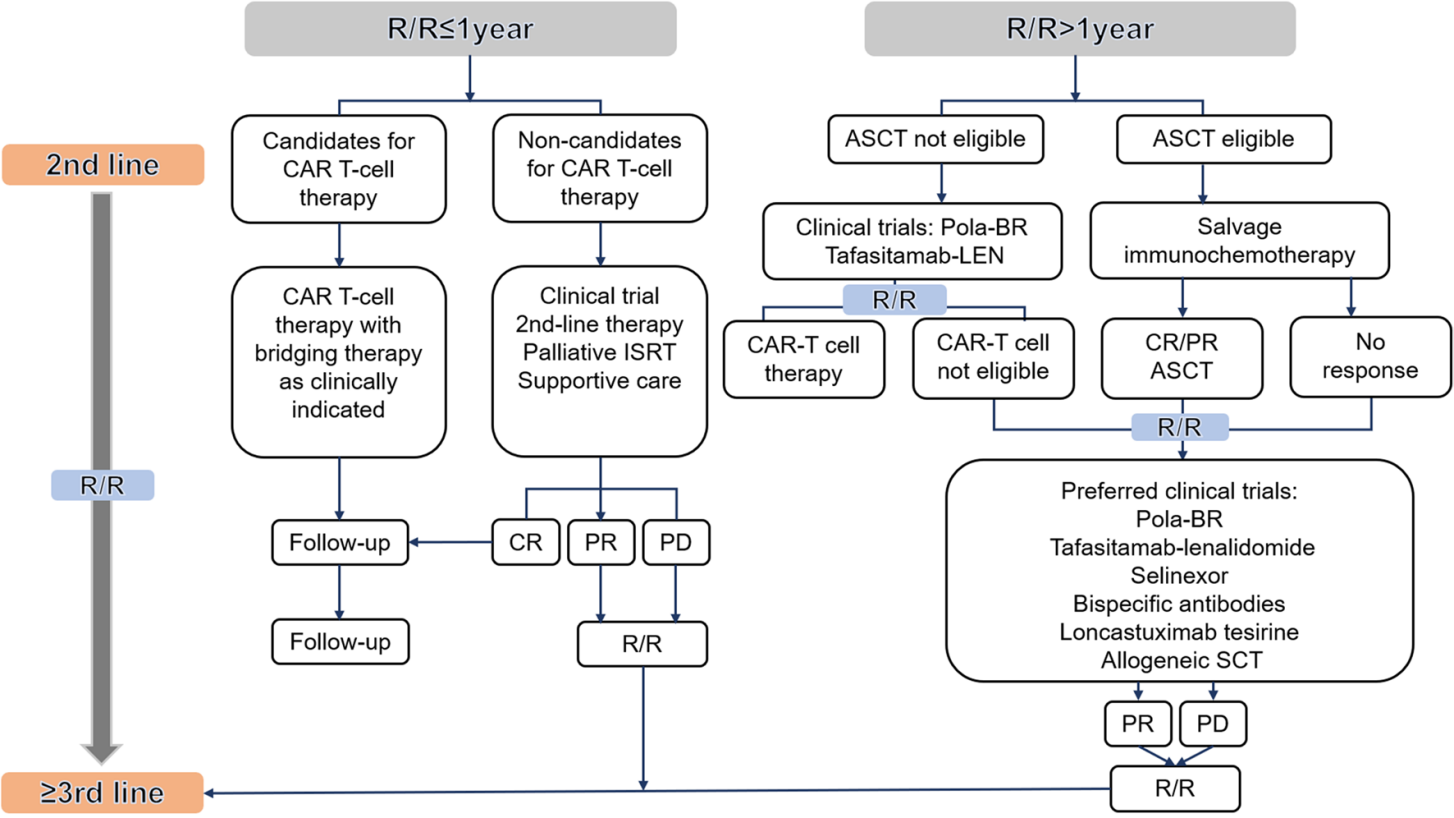
Algorithm for the treatment of relapsed/refractory diffuse large B-cell lymphoma patients

Clinical trials are first recommended for relapsed or refractory (R/R) DLBCL. For patients who R/R to their first-line therapy, salvage high-dose chemotherapy and an autologous stem-cell transplant (ASCT) are the standard second-line treatments [161, 162] (Fig. 3). The strategy, however, is beneficial only to healthy patients without comorbidities [161]. Furthermore, studies have shown that even intensive therapy can fail to improve the outcome of patients with primary refractory disease or patients who are relapsing within 12 months of first-line therapy, with an objective response rate (ORR) of 26%, a CR rate of 7%, and a median OS rate of 6.3 months were achieved [163].

#### CAR t-cell therapy

**Fig. 4:**
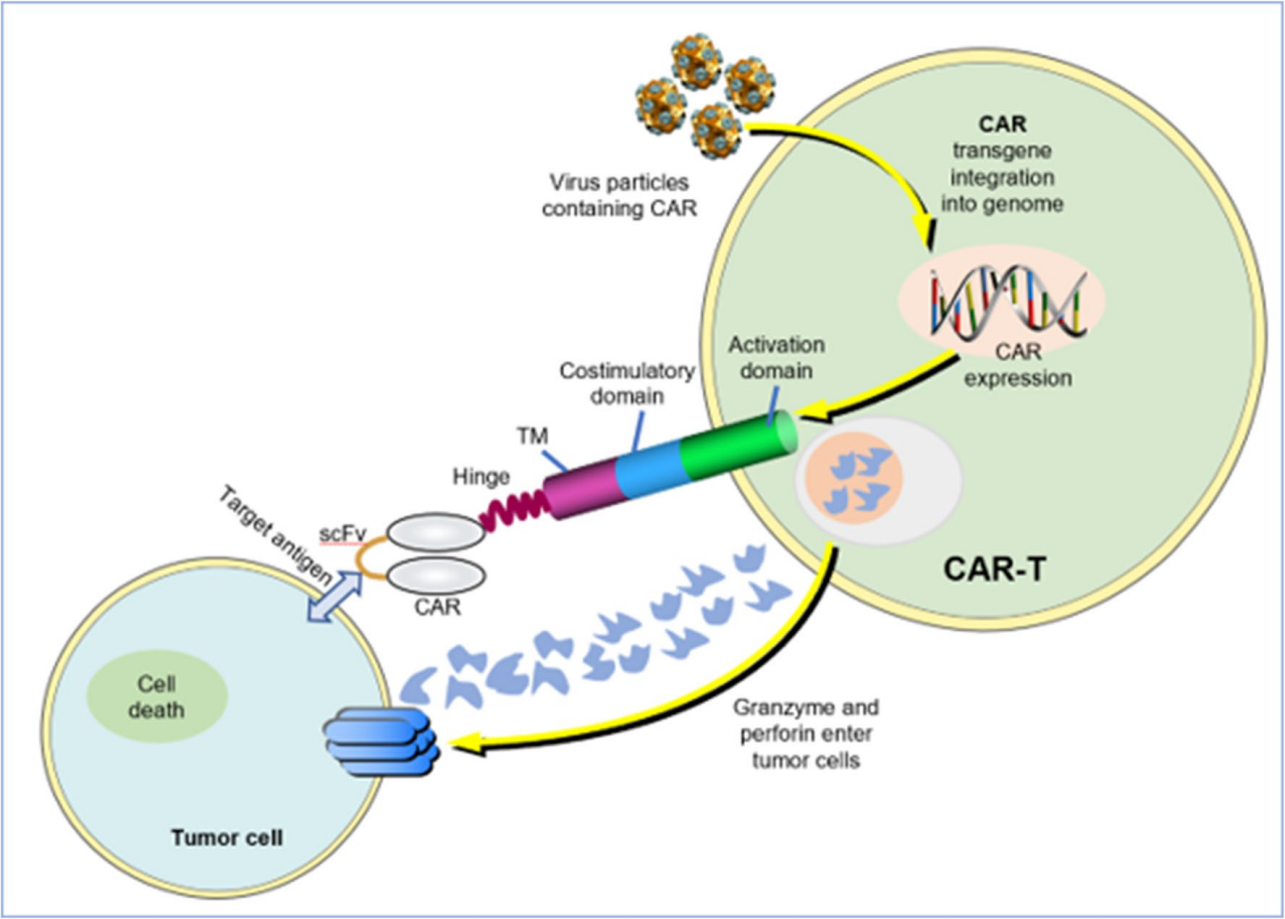
Targeting antigen-expressing tumor cells with CAR T cells. T cells transduced with viral particles harboring the CAR-encoding transgene express CARs on their surfaces in a stable manner. The activation of CAR-T cells occurs when they encounter a tumor antigen, releasing perforin and granzymes that cause the tumor cells to die

Newly authorized treatment choices, like chimeric antigen receptor T cell (CAR-T) therapy, have been recently approved. Polatuzumab vedotin, tafasitamab in combination with lenalidomide, loncastuximab tesirine, or selinexor could be potential treatment choices for individuals with R/R DLBCL, particularly for those who have undergone two or more lines of therapy (LOTs) and/or are not suitable candidates for autologous stem cell transplantation (ASCT) [164–167]. Clinical trials of CAR-T in phase 1/2 reported an ORR of 52 to 82% [168–170]. More recently, clinical trials testing CAR-T therapies against salvage therapy with the intention of combining with HDT-ASCT have demonstrated significant benefits among patients suffering from primary refractory DLBCL or who have relapsed within 12 months of receiving 1line therapy, this represents an important step forward for patients with R/R DLBCL [171]. Although CAR-T therapy may be effective for some patients, it has been plagued with serious toxicities, high rates of disease progression, and limited eligibility for treatment [172, 173]. In patients with R/R cancer, CAR T-cell therapy is a superior treatment option. CD19 is the first approved product that involves autologous T cells. In early clinical trials, the overall response and CR rates of relapsed and refractory patients after treatment with axicabtagene ciloleucel, tisagenlecleucel, and lisocabtagene maraleucel were 52 to 82% and 40 to 54%, respectively [168–170]. In subsequent studies, 37% of patients had a median survival of 27 months after receiving axicabtagene ciloleucel [174]. Of course, there are errors in the experimental results because patients receiving treatment are all selected. Because of its side effects, CAR T-cell therapy is not suitable for all patients. The investigators found that, after the patients received CAR T-cell therapy, the incidence of grade 3–4 cytokine release syndrome and neurotoxic effects was 2–22% and 10–28%, respectively [168–170]. At present, the wide use of CAR T-cell therapy is limited by various factors, such as large toxicity and side effects, high economic costs and the disease process of patients [175]. Therefore, it is urgent to develop multitarget and allogeneic off-the-shelf products to provide more choices for patients in the future. Figure 4 shows the pattern diagram of CAR-Ts. Some small molecule targeted drugs, such as ABT-199, a selective inhibitor of BCL-2, lenalidomide, a tyrosine kinase inhibitor, and an epigenetic regulator (EZH2 inhibitor tazemetostat), have been applied in the clinic as an important part of the combined treatment regimen [151, 176–178]. In addition, pathway-based approaches should be taken seriously, such as NOTCH, JAK-STAT, and PI3K-AKT-mTOR [179]. The novel perspectives and breakthroughs in the treatment of DLBCL are listed in Table 5.

**Table 5:** New drugs and the main mechanism

New drugs	Types	Mechanism	Ref
polatuzumab vedotin	antibody–drug conjugate	Target cells that expressed CD79b and function on the microtubule to accelerate apoptosis	[180]
Loncastuximab tesirine	antibody–drug conjugate	Conjugate anti-CD19 antibody and Alkylated drugs and function in B cell lymphoma	[181, 182]
Selinexor	XPO1 inhibitor	Inhibit XPO1 which over- expressed in DLBCL cells	[183]
Tafasitamab	monoclonal antibody	Mediate antibody-dependent cytotoxicity and phagocytosis targeting on CD19 positive cells	[184]
Axicabtagene ciloleucel	CAR-T	Function on CD19 positive lymphoma cells	[185]
Tisagenlecleucel	CAR-T	Function on CD19 positive lymphoma cells	[169]
Lisocabtagene maraleucel	CAR-T	Function on CD19 positive lymphoma cells	[186]
Glofitamab	Bispecific antibody	Engage and exterminate B cells by bispecifically targeting CD3 and CD20	[187]
Epcoritamab	Bispecific antibody	Engage and exterminate B cells by bispecifically targeting CD3 and CD20	[188]

XPO1: exportin 1; CAR-T: chimeric antigen receptor T cell immunotherapy

#### Checkpoint inhibitors

1. Cytotoxic T lymphocyte-associated antigen-4 (CTLA-4)
  Both CD4^+^ and CD8^+^ T cells express the homologous receptors CD28 and CTLA4. Activation of T cells is mediated by the opposing effects of these receptors. The T-cell-mediated immune response is activated by CD28, while the T-cell-mediated immune response is suppressed by CTLA-4. Ipilimumab, the first anti-CTLA-4 monotherapy has achieved significant clinical effects since in 2011. The most striking observation regarding ipilimumab was the increase in overall patient survival of up to 10 years for some patients [189, 190].
2. Programmed cell death (PD-1)
  There is a 20% sequence homology between PD-1, which is also known as CD279, and CTLA4, which was discovered in 1992. As an inhibitor of both adaptive and innate immune responses. In addition, PD-1 has sustained expression during persistent antigen encounters, which limits protective immunity. T cells are not the only cells expressing PD-1 during persistent antigen encounters, and the phenomenon can be observed both in hematopoietic and nonhematopoietic cells. Thus, PD-1 plays an important role in secondary lymphoid organ immune cell function [191, 192].

#### Next-generation immune checkpoint targets

It is expected that an increasing number of immune checkpoint targets will be developed as medical technology advances, including LAG-3 (CD223), B7-H3 and B7-H4, A2aR and CD73, and NKG2A.

## Conclusions

In this review, we summarize the genetic events of DLBCL and how they promote the development of this type of lymphoma and discuss the clinical importance of genetic abnormalities. The application of genetics, immunology and TME in the classification, diagnosis and treatment of DLBCL is helpful to better understand the biology of lymphoma. Several elegant studies have uncovered the functional implications of genetic aberrations, including those involving BCL-6, CREBBP, KMT2D and others. However, the exact functional relevance of many genetic aberrations remains unclear. There is limited information available at present regarding the stages of B-cell maturation during which these aberrations occur. Genetic and pathway mutations recurrent in DLBCL reveal vulnerabilities in lymphoma cells that are often associated with distinct lymphoma subtypes, and more effective, targeted therapeutic approaches could be developed. The findings from these studies are already being applied to the development of products, services and novel drugs or drug combinations being tested (or repositioned) in DLBCL to combat specific dysregulated program. The different diagnostic criteria of DLBCL are described in detail. Finally, the treatment progress of DLBCL was summarized. The latest description of the genetics, biology and diagnostics of DLBCL will help to develop new and, more importantly, accurate treatment methods for patients with DLBCL.

## Data Availability

Not applicable.

## Abbreviations

DLBCLs: Diffuse large B-cell lymphomas
GEP: Gene-expression profile
ABC: Activated B-cell
GCB: Germinal-center B-cell
R-CHOP: Rituximab Cyclophosphamide, Doxorubicin, Vincristine, Prednisone; COO: Cell-of-origin
LME: Lymphoma microenvironment
RT: Radiotherapy
BCL-2: B-cell lymphoma-2
CAR-T: Chimeric antigen receptor T cell immunotherapy
NHL: Non-Hodgkin lymphoma
JAK: Janus kinase
NF-Κb: Nuclear factor-kB
MYD88: Myeloid differentiation primary response 88
PFS: Progression-free survival
STAT3: Signal transducer and activator of transcription 3
BCR: B-cell receptor
BTK: Bruton tyrosine kinase
NK cell: Natural killer cell
scFv: Single-chain variable fragment
TM: Transmembrane domain
CNS: Central nervous system
DA-EPOCH-CHOP: Dose-adjusted-etoposide, Prednisone, Vincristine, Cyclophosphamide and Doxorubicin- Cyclophosphamide, Doxorubicin, Vincristine and Prednisone
XPO1: Exportin 1
ALK: Anaplastic lymphoma kinase
IRF4: Interferon regulatory factor 4
BCL-6: B-cell lymphoma-6
NOS: Not otherwise specified
PMBL: Primary mediastinal large B-cell lymphoma
FAD: Food drug administration
OSR: 5- Or 3-year overall survival rate
EFS: Event-free survival
ASCT: Autologous stem-cell transplant
CR/PR: Complete remission/partial remission
ORR: Objective response rat
CR: Complete remission
OS: Overall survival
